# Crystal structures of the two isomeric hydrogen-bonded cocrystals 2-chloro-4-nitro­benzoic acid–5-nitro­quinoline (1/1) and 5-chloro-2-nitro­benzoic acid–5-nitro­quinoline (1/1)

**DOI:** 10.1107/S2056989019013896

**Published:** 2019-10-22

**Authors:** Kazuma Gotoh, Hiroyuki Ishida

**Affiliations:** a Department of Chemistry, Faculty of Science, Okayama University, Okayama 700-8530, Japan

**Keywords:** crystal structure, 2-chloro-4-nitro­benzoic acid, 5-chloro-2-nitro­benzoic acid, 5-nitro­quinoline, hydrogen bond, Hirshfeld surface

## Abstract

The structures of two isomeric com­pounds of 5-nitro­quinoline with chloro- and nitro-substituted benzoic acid, namely, 2-chloro-4-nitro­benzoic acid–5-nitro­quinoline (1/1), (I), and 5-chloro-2-nitro­benzoic acid–5-nitro­quinoline (1/1), (II), both C_7_H_4_ClNO_4_·C_9_H_6_N_2_O_2_, have been determined at 190 K. In each com­pound, the acid and base mol­ecules are held together by an O—H⋯N hydrogen bond. In the crystal of (I), the hydrogen-bonded acid–base units are linked by a C—H⋯O hydrogen bond, forming a tape structure along [10]. The tapes are stacked into a layer parallel to the *ab* plane *via* N—O⋯π inter­actions between the nitro group of the base mol­ecule and the quinoline ring system. The layers are further linked by other C—H⋯O hydrogen bonds, forming a three-dimensional network. In the crystal of (II), the hydrogen-bonded acid–base units are linked into a wide ribbon structure running along [10] *via* C—H⋯O hydrogen bonds. The ribbons are further linked *via* another C—H⋯O hydrogen bond, forming a layer parallel to (110). Weak π–π inter­actions [centroid–centroid distances of 3.7080 (10) and 3.7543 (9) Å] are observed between the quinoline ring systems of adjacent layers. Hirshfeld surfaces for the 5-nitro­quinoline mol­ecules of the two com­pounds mapped over shape index and *d*_norm_ were generated to visualize the weak inter­molecular inter­actions.

## Abstract 2

The structures of the two isomeric hydrogen-bonded 1:1 cocrystals of 5-nitro­quinoline with 2-chloro-4-nitro­benzoic acid and 5-chloro-2-nitro­benzoic acid have been determined at 190 K. In each crystal, the acid and base mol­ecules are linked by a short O—H⋯N hydrogen bond.

## Chemical context   

The properties of hydrogen bonds formed between organic acids and organic bases depend on the p*K*_*a*_ values of the acids and bases, as well as the inter­molecular inter­actions in the crystals. For the system of quinoline and chloro- and nitro-substituted benzoic acids, we have shown that three com­pounds of quinoline with 3-chloro-2-nitro­benzoic acid, 4-chloro-2-nitro­benzoic acid and 5-chloro-2-nitorbenzoic acid, the Δp*K*_*a*_ [p*K*_*a*_(base) – p*K*_*a*_(acid)] values of which are 3.08, 2.93 and 3.04, respectively, have a short double-well O⋯H⋯N hydrogen bond between the carb­oxy O atom and the aromatic N atom (Gotoh & Ishida, 2009 ▸). Similar O⋯H⋯N hydrogen bonds have also been observed in com­pounds of phthalazine with 3-chloro-2-nitro­benzoic acid and 4-chloro-2-nitrobenzoic acid with Δp*K*_*a*_ values of 1.65 and 1.50, respectively (Gotoh & Ishida, 2011*a* ▸), and of iso­quinoline with 3-chloro-2-nitro­benzoic acid with Δp*K*_*a*_ = 3.58 (Gotoh & Ishida, 2015 ▸). On the other hand, in 2-chloro-4-nitro­benzoic acid–quinoline (1/1) with Δp*K*_*a*_ = 2.86 (Gotoh & Ishida, 2011*b* ▸), 3-chloro-2-nitro­benzoic acid–5-nitro­quinoline (1/1) with Δp*K*_*a*_ = 0.98, 3-chloro-2-nitro­benzoic acid–6-nitro­quinolune (1/1) with Δp*K*_*a*_ = 1.42 and 8-hy­droxy­quinolinium 3-chloro-2-nitro­benzoate with Δp*K*_*a*_ = 3.02 (Gotoh & Ishida, 2019 ▸), such a short disordered hydrogen bond was not observed, suggesting that the strength of the hydrogen bond between the acid O atom and the base N atom is strongly influenced by other weak inter­molecular inter­actions.
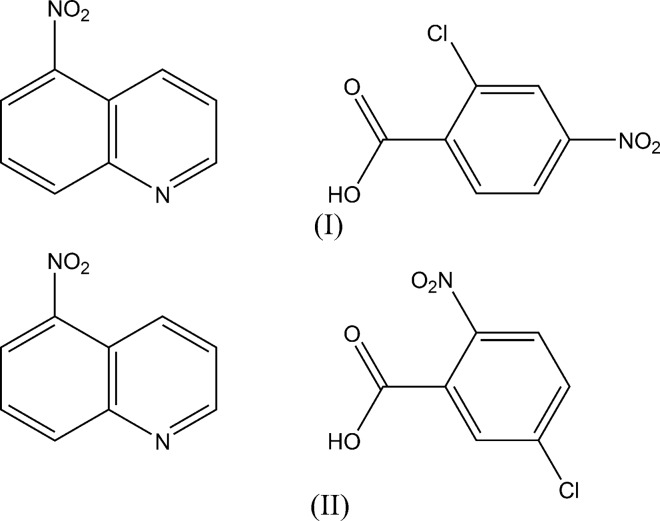


We report here the crystal structures of the isomeric com­pounds 2-chloro-4-nitro­benzoic acid–5-nitro­quinoline (1/1) (Δp*K*_*a*_ = 0.76) and 5-chloro-2-nitro­benzoic acid–5-nitro­quinoline (1/1) (Δp*K*_*a*_ = 0.94), in order to extend our studies of short hydrogen bonding and weak inter­molecular inter­actions in the quinoline derivative–chloro- and nitro-substituted benzoic acid system.

## Structural commentary   

**Figure 1:**
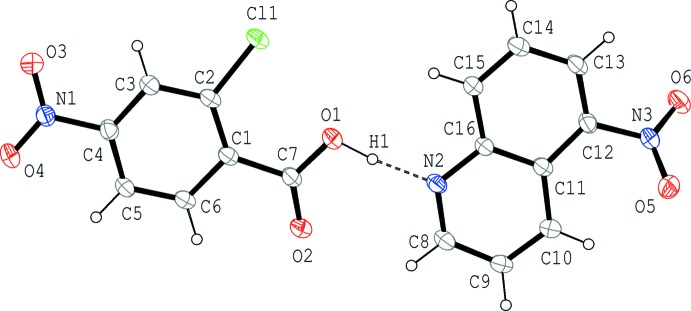
The mol­ecular structure of (I), showing the atom-numbering scheme. Displacement ellipsoids are drawn at the 50% probability level and H atoms are shown as small spheres of arbitrary radii. The O—H⋯N hydrogen bond is indicated by a dashed line.

Compound (I) crystallizes in the noncentrosymmetric space group *P*2_1_, where the acid and base mol­ecules are held together by an O—H⋯N hydrogen bond between the carb­oxy group and the N atom of the base (Fig. 1 ▸ and Table 1 ▸). The hydrogen-bonded acid–base unit is approximately planar; the quinoline ring system (N2/C8–C16) makes dihedral angles of 3.94 (17) and 7.5 (5)°, respectively, with the benzene ring (C1–C6) and the carb­oxy group (O1/C7/O2). In the acid mol­ecule, the benzene ring makes dihedral angles of 4.3 (5) and 2.5 (5)°, respectively, with the carb­oxy group and the nitro group (O3/N1/O4), while in the base mol­ecule, the quinoline ring system and the attached nitro group (O5/N3/O6) are somewhat twisted with a dihedral angle of 36.2 (5)°.

**Figure 2:**
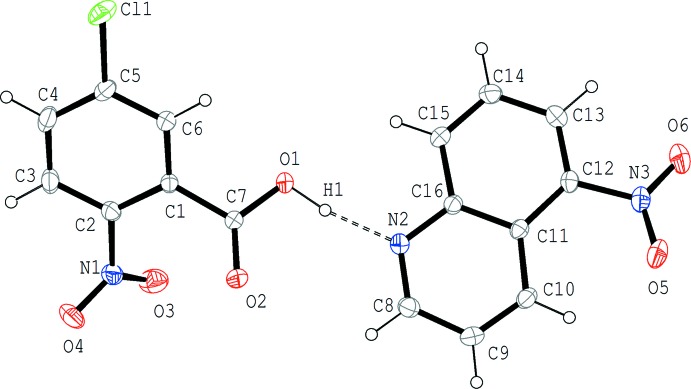
The mol­ecular structure of (II), showing the atom-numbering scheme. Displacement ellipsoids are drawn at the 50% probability level and H atoms are shown as small spheres of arbitrary radii. The O—H⋯N hydrogen bond is indicated by a dashed line.

The mol­ecular structure of (II) is shown in Fig. 2 ▸. Similar to (I), the acid and base mol­ecules are held together by an O—H⋯N hydrogen bond (Table 2 ▸). In the acid–base unit, the quinoline ring system and the hydrogen-bonded carb­oxy group are almost coplanar, with a dihedral angle of 2.9 (2)°, while the quinoline ring system and the benzene ring of the acid are twisted with respect to each other by a dihedral angle of 37.37 (6)°. In the acid mol­ecule, the benzene ring makes dihedral angles of 40.3 (2) and 47.12 (19)°, respectively, with the carb­oxy and nitro groups. In the base mol­ecule, the dihedral angle between the quinoline ring system and the attached nitro group is 11.3 (2)°.

## Supra­molecular features   

**Figure 3:**
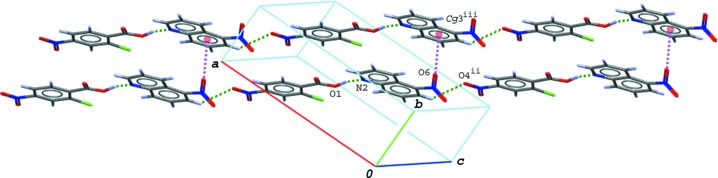
A packing diagram of (I), showing the hydrogen-bonded tape structure formed *via* O—H⋯N and C—H⋯O hydrogen bonds (green dashed lines), and N—O⋯π inter­actions (magenta dashed lines) between the tapes. The N—O⋯π inter­actions including the centroid of the ten-membered quinoline ring system (*Cg*4) have been omitted for clarity. [Symmetry codes: (ii) *x* − 1, *y* + 2, *z*; (iii) *x*, *y* + 1, −*z* + 1.]

In the crystal of (I), the hydrogen-bonded acid–base units are linked by a C—H⋯O hydrogen bond (C13—H13⋯O4^ii^; symmetry code as in Table 1 ▸), forming a tape structure along [1
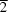
0]. The tapes are stacked into a layer parallel to the *ab* plane (Fig. 3 ▸) *via* N—O⋯π contacts (N3—O5⋯*Cg*3^iii^ and N3—O5⋯*Cg*4^iii^; Table 1 ▸) between the nitro group of the base and the quinoline ring system; *Cg*3 and *Cg*4 are the centroids of the C11–C16 ring and the N2/C8–C16 ring system of the base mol­ecule, respectively. The layers are further linked by other C—H⋯O hydrogen bonds (C8—H8⋯O2^i^ and C9—H9⋯O2^i^; Table 1 ▸), forming a three-dimensional network.

**Figure 4:**
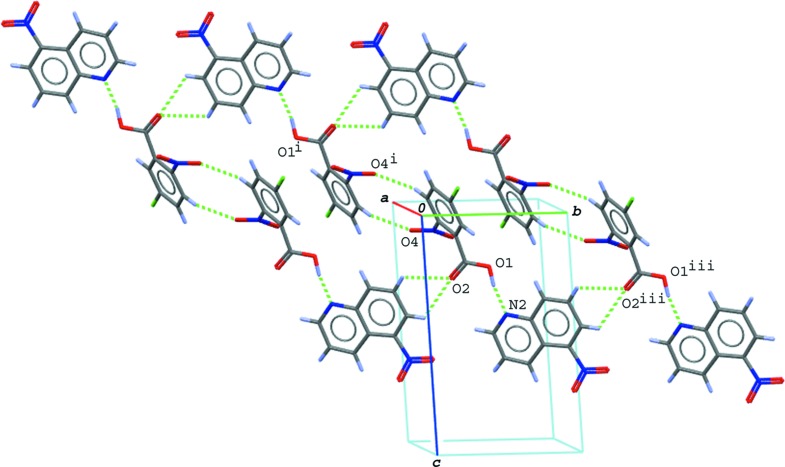
A packing diagram of (II), showing the wide ribbon structure running along [1
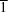
0] formed by O—H⋯N and C—H⋯O hydrogen bonds (green dashed lines). [Symmetry codes: (i) −*x* + 2, −*y*, −*z*; (iii) *x* − 1, *y* + 1, *z*.]

In the crystal of (II), the hydrogen-bonded acid–base units are linked into a wide ribbon structure running along [1
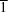
0] (Fig. 4 ▸) *via* C—H⋯O hydrogen bonds (C3—H3⋯O4^i^, C13—H13⋯O2^iii^ and C14—H14⋯O2^iii^; symmetry codes as in Table 2 ▸); the mean plane of the non-H atoms in the ribbon is parallel to (773). The ribbons are further linked *via* another C—H⋯O hydrogen bond (C10—H10⋯O3^ii^; Table 2 ▸), forming a layer parallel to (110). Between the layers, weak π–π inter­actions are observed; the centroid–centroid distances are 3.7080 (10) and 3.7543 (9) Å, respectively, for *Cg*2⋯*Cg*2^iv^ and *Cg*2⋯*Cg*4^vi^, where *Cg*2 and *Cg*4 are the centroids of the N2/C8–C11/C16 ring and the N2/C8–C16 ring system of the base mol­ecule, respectively [symmetry code: (iv) −*x*, −*y* + 1, −*z* + 1].

**Figure 5:**
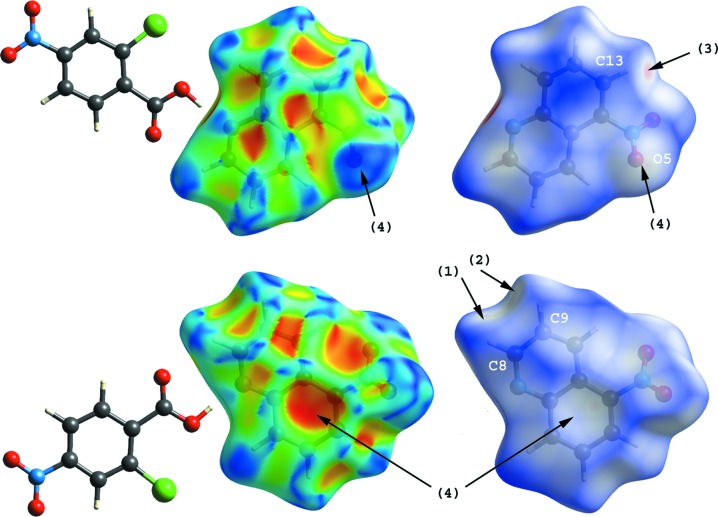
Hirshfeld surfaces [front (top) and back (bottom) views] for the 5-nitro­quinoline mol­ecule of (I) mapped over shape index and *d*_norm_, indicating the C—H⋯O [arrows (1)–(3)] and N—O⋯π [arrows (4)] inter­actions.

**Figure 6:**
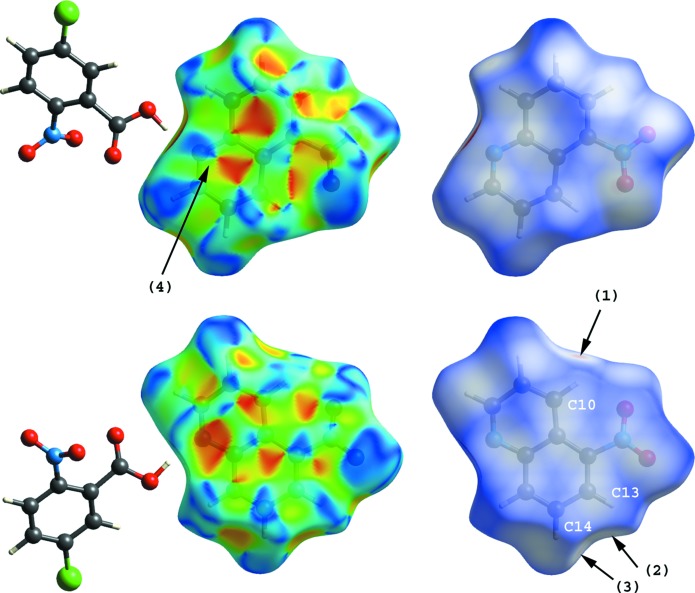
Hirshfeld surfaces [front (top) and back (bottom) views] for the 5-nitro­quinoline mol­ecule of (II) mapped over shape index and *d*_norm_, indicating the C—H⋯O [arrows (1)–(3)] and π–π [arrow (4)] inter­actions.

Hirshfeld surfaces for the 5-nitro­quinoline mol­ecules of (I) and (II), mapped over shape index and *d*_norm_ (Turner *et al.*, 2017 ▸; McKinnon *et al.*, 2004 ▸, 2007 ▸), are shown in Figs. 5 ▸ and 6 ▸. The three C—H⋯O inter­actions in (I) (C8—H8⋯O2^i^, C9—H9⋯O2^i^ and C13—H13⋯O4^ii^; Table 1 ▸) are viewed as faint-red spots on the *d*_norm_ surfaces [arrows (1)–(3); Fig. 5 ▸]. In addition to these inter­actions, the N—O⋯π contacts (N3—O5⋯*Cg*3^iii^ and N3—O5⋯*Cg*4^iii^; Table 1 ▸) are shown as broad blue and red regions, respectively, in the front and back views of shape-index surfaces [arrows (4)]. The three C—H⋯O inter­actions in (II) (C10—H10⋯O3^ii^, C13—H13⋯O2^iii^ and C14—H14⋯O2^iii^; Table 2 ▸) are also represented as faint-red spots on the *d*_norm_ surfaces [arrows (1)–(3); Fig. 6 ▸]. By contrast with the shape-index surfaces of (I), π–π inter­actions between the quinoline ring systems of inversion-related mol­ecules [*Cg*2⋯*Cg*2^iv^ and *Cg*2⋯*Cg*4^vi^; symmetry code: (iv) −*x*, −*y* + 1, −*z* + 1] are indicated by blue and red triangles on the shape-index surface [arrow (4) in the front view of (II)].

## Database survey   

A search of the Cambridge Structural Database (Version 5.40, last update August 2019; Groom *et al.*, 2016 ▸) for organic cocrystals/salts of 5-nitro­quinoline with carb­oxy­lic acid derivatives gave five structures, namely, 3-amino­benzoic acid–5-nitro­quinoline (1/1) (refcode PANYIM; Lynch *et al.*, 1997 ▸), 4-animo­benzoic acid–5-nitro­quinoline (1/2) (PANZEJ; Lynch *et al.*, 1997 ▸), indole-2-carb­oxy­lic acid–5-nitro­quinoline (1/2) (GISGUK; Lynch *et al.*, 1998 ▸), indole-3-acetic acid–5-nitro­quinoline (1/2) (GISHAR: Lynch *et al.*, 1998 ▸) and (2,4,5-tri­chloro­phen­oxy)acetic acid–5-nitro­quinoline (1/1) (XAP­WOA; Lynch *et al.*, 1999 ▸). In these com­pounds, the dihedral angles between the quinoline ring system and the attached nitro group vary in the wide range 2.2 (4)–32.9 (4)°, which implies that the orientation of the nitro group is mainly affected by inter­molecular inter­actions.

A search for organic cocrystals/salts of 2-chloro-4-nitro­benzoic acid with base mol­ecules gave 60 structures, while for organic cocrystals/salts of 5-chloro-2-nitro­benzoic acid with base mol­ecules, five com­pounds were reported. Limiting the search to quinoline derivatives of these com­pounds gave three com­pounds, namely, 2-chloro-4-nitro­benzoic acid–quinoline (1/1) (YAGFAP; Gotoh & Ishida, 2011*b* ▸), 8-hy­droxy­quinolinium 2-chloro-4-nitro­benzoate (WOPDEM; Babu & Chandrasekaran, 2014 ▸) and 5-chloro-2-nitro­benzoic acid–quinoline (1/1) (AJIXAT; Gotoh & Ishida, 2009 ▸).

## Synthesis and crystallization   

Crystals of com­pounds (I) and (II) were obtained by slow evaporation from aceto­nitrile solutions of 5-nitro­quinoline with chloro­nitro­benzoic acids in a 1:1 molar ratio at room temperature [80 ml aceto­nitrile solution of 5-nitro­quinoline (0.117 g) and 2-chloro-4-nitro­benzoic acid (0.135 g) for (I), and 50 ml aceto­nitrile solution of 5-nitro­quinoline (0.099 g) and 5-chloro-2-nitro­benzoic acid (0.112 g) for (II)].

## Refinement   

Crystal data, data collection, and structure refinement details are summarized in Table 3 ▸. All H atoms in com­pounds (I) and (II) were found in difference Fourier maps. H atoms on O atoms in (I) and (II) were refined freely, with distances of O1—H1 = 1.02 (8) Å in (I) and O1—H1 = 0.99 (4) Å in (II). Other H atoms were positioned geometrically (C—H = 0.95 Å) and treated as riding, with *U*_iso_(H) = 1.2*U*_eq_(C).

## Supplementary Material

Crystal structure: contains datablock(s) global, I, II. DOI: 10.1107/S2056989019013896/lh5931sup1.cif

Structure factors: contains datablock(s) I. DOI: 10.1107/S2056989019013896/lh5931Isup2.hkl

Structure factors: contains datablock(s) II. DOI: 10.1107/S2056989019013896/lh5931IIsup3.hkl

CCDC references: 1958672, 1958673, 1958672, 1958673

Additional supporting information:  crystallographic information; 3D view; checkCIF report

## Figures and Tables

**Table 1:** Hydrogen-bond geometry (Å, °) for (I) *Cg*3 and *Cg*4 are the centroids of the C11–C16 ring and the N2/C8–C16 ring system, respectively.

*D*—H⋯*A*	*D*—H	H⋯*A*	*D*⋯*A*	*D*—H⋯*A*
O1—H1⋯N2	1.02 (8)	1.58 (7)	2.585 (5)	168 (7)
C8—H8⋯O2^i^	0.95	2.59	3.174 (6)	120
C9—H9⋯O2^i^	0.95	2.56	3.152 (6)	120
C13—H13⋯O4^ii^	0.95	2.52	3.289 (6)	138
N3—O5⋯*Cg*3^iii^	1.23 (1)	3.06 (1)	3.724 (4)	113 (1)
N3—O5⋯*Cg*4^iii^	1.23 (1)	3.25 (1)	4.118 (4)	128 (1)

Symmetry codes: (i) 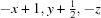; (ii) 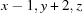; (iii) 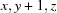.

**Table 2:** Hydrogen-bond geometry (Å, °) for (II)

*D*—H⋯*A*	*D*—H	H⋯*A*	*D*⋯*A*	*D*—H⋯*A*
O1—H1⋯N2	0.99 (4)	1.66 (4)	2.6405 (17)	169 (3)
C3—H3⋯O4^i^	0.95	2.49	3.408 (3)	162
C10—H10⋯O3^ii^	0.95	2.54	3.254 (2)	132
C13—H13⋯O2^iii^	0.95	2.59	3.190 (2)	121
C14—H14⋯O2^iii^	0.95	2.56	3.173 (2)	122

Symmetry codes: (i) 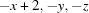; (ii) 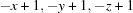; (iii) 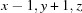.

**Table 3:** Experimental details

	(I)	(II)
Crystal data		
Chemical formula	C_7_H_4_ClNO_4_·C_9_H_6_N_2_O_2_	C_7_H_4_ClNO_4_·C_9_H_6_N_2_O_2_
*M*_r_	375.72	375.72
Crystal system, space group	Monoclinic, *P*2_1_	Triclinic, *P* 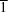
Temperature (K)	190	190
*a*, *b*, *c* (Å)	12.8265 (13), 4.7699 (5), 13.5033 (16)	7.6682 (6), 8.6515 (8), 12.8609 (10)
α, β, γ (°)	90, 109.713 (3), 90	79.170 (3), 78.968 (2), 70.394 (3)
*V* (Å^3^)	777.73 (15)	781.80 (11)
*Z*	2	2
Radiation type	Mo *K*α	Mo *K*α
μ (mm^−1^)	0.29	0.29
Crystal size (mm)	0.37 × 0.18 × 0.10	0.26 × 0.20 × 0.18

Data collection		
Diffractometer	Rigaku R-AXIS RAPID	Rigaku R-AXIS RAPID
Absorption correction	Numerical (*NUMABS*; Higashi, 1999 ▸)	Numerical (*NUMABS*; Higashi, 1999 ▸)
*T*_min_, *T*_max_	0.913, 0.972	0.933, 0.950
No. of measured, independent and observed [*I* > 2σ(*I*)] reflections	14435, 4168, 2859	9772, 4502, 3075
*R*_int_	0.058	0.055
(sin θ/λ)_max_ (Å^−1^)	0.703	0.703

Refinement		
*R*[*F* ^2^ > 2σ(*F* ^2^)], *wR*(*F* ^2^), *S*	0.056, 0.169, 1.05	0.052, 0.148, 1.09
No. of reflections	4168	4502
No. of parameters	239	239
No. of restraints	1	0
H-atom treatment	H atoms treated by a mixture of independent and constrained refinement	H atoms treated by a mixture of independent and constrained refinement
Δρ_max_, Δρ_min_ (e Å^−3^)	0.34, −0.65	0.42, −0.39
Absolute structure	Flack *x* determined using 898 quotients [(*I* ^+^) − (*I* ^−^)]/[(*I* ^+^) + (*I* ^−^)] (Parsons *et al.*, 2013 ▸)	–
Absolute structure parameter	0.01 (6)	–

Computer programs: *PROCESS-AUTO* (Rigaku, 2006 ▸), *SHELXT2018* (Sheldrick, 2015*a* ▸), *SHELXL2018* (Sheldrick, 2015*b* ▸), *ORTEP-3 for Windows* (Farrugia, 2012 ▸), *Mercury* (Macrae *et al.*, 2008 ▸), *CrystalStructure* (Rigaku, 2018 ▸) and *PLATON* (Spek, 2015 ▸).

## supplementary crystallographic information

### 2-Chloro-4-nitrobenzoic acid–5-nitroquinoline (I) Crystal data

**Table:**

C_7_H_4_ClNO_4_·C_9_H_6_N_2_O_2_	*F*(000) = 384.00
*M**_r_* = 375.72	*D*_x_ = 1.604 Mg m^−^^3^
Monoclinic, *P*2_1_	Mo *K*α radiation, λ = 0.71075 Å
*a* = 12.8265 (13) Å	Cell parameters from 11483 reflections
*b* = 4.7699 (5) Å	θ = 3.1–30.0°
*c* = 13.5033 (16) Å	µ = 0.29 mm^−^^1^
β = 109.713 (3)°	*T* = 190 K
*V* = 777.73 (15) Å^3^	Block, colorless
*Z* = 2	0.37 × 0.18 × 0.10 mm

### 2-Chloro-4-nitrobenzoic acid–5-nitroquinoline (I) Data collection

**Table:**

Rigaku R-AXIS RAPID diffractometer	2859 reflections with *I* > 2σ(*I*)
Detector resolution: 10.000 pixels mm^-1^	*R*_int_ = 0.058
ω scans	θ_max_ = 30.0°, θ_min_ = 3.1°
Absorption correction: numerical (NUMABS; Higashi, 1999)	*h* = −17→18
*T*_min_ = 0.913, *T*_max_ = 0.972	*k* = −6→6
14435 measured reflections	*l* = −18→18
4168 independent reflections	

### 2-Chloro-4-nitrobenzoic acid–5-nitroquinoline (I) Refinement

**Table:**

Refinement on *F*^2^	Secondary atom site location: difference Fourier map
Least-squares matrix: full	Hydrogen site location: mixed
*R*[*F*^2^ > 2σ(*F*^2^)] = 0.056	H atoms treated by a mixture of independent and constrained refinement
*wR*(*F*^2^) = 0.169	*w* = 1/[σ^2^(*F*_o_^2^) + (0.0939*P*)^2^] where *P* = (*F*_o_^2^ + 2*F*_c_^2^)/3
*S* = 1.05	(Δ/σ)_max_ < 0.001
4168 reflections	Δρ_max_ = 0.34 e Å^−^^3^
239 parameters	Δρ_min_ = −0.64 e Å^−^^3^
1 restraint	Absolute structure: Flack *x* determined using 898 quotients [(I+)-(I-)]/[(I+)+(I-)] (Parsons *et al.*, 2013)
Primary atom site location: structure-invariant direct methods	Absolute structure parameter: 0.01 (6)

### 2-Chloro-4-nitrobenzoic acid–5-nitroquinoline (I) Special details

**Table:**

Geometry. All esds (except the esd in the dihedral angle between two l.s. planes) are estimated using the full covariance matrix. The cell esds are taken into account individually in the estimation of esds in distances, angles and torsion angles; correlations between esds in cell parameters are only used when they are defined by crystal symmetry. An approximate (isotropic) treatment of cell esds is used for estimating esds involving l.s. planes.

### 2-Chloro-4-nitrobenzoic acid–5-nitroquinoline (I) Fractional atomic coordinates and isotropic or equivalent isotropic displacement parameters (Å^2^)

**Table:**

	*x*	*y*	*z*	*U*_iso_*/*U*_eq_	
Cl1	0.57519 (8)	0.0684 (3)	0.43612 (8)	0.0493 (3)	
O1	0.5077 (3)	0.4634 (8)	0.2687 (3)	0.0477 (8)	
O2	0.5751 (3)	0.4867 (9)	0.1386 (3)	0.0558 (10)	
O3	0.9097 (3)	−0.5701 (8)	0.4815 (3)	0.0521 (8)	
O4	0.9774 (2)	−0.4811 (7)	0.3586 (3)	0.0484 (8)	
O5	0.0470 (2)	1.5716 (7)	0.0857 (3)	0.0485 (7)	
O6	−0.0635 (2)	1.3007 (9)	0.1321 (3)	0.0555 (9)	
N1	0.9100 (3)	−0.4421 (8)	0.4027 (3)	0.0383 (7)	
N2	0.3733 (3)	0.8494 (8)	0.1655 (3)	0.0372 (8)	
N3	0.0279 (3)	1.3624 (8)	0.1297 (3)	0.0410 (8)	
C1	0.6593 (3)	0.1676 (9)	0.2761 (3)	0.0338 (8)	
C2	0.6664 (3)	0.0245 (9)	0.3678 (3)	0.0369 (9)	
C3	0.7492 (3)	−0.1771 (10)	0.4099 (3)	0.0381 (9)	
H3	0.753753	−0.276100	0.472305	0.046*	
C4	0.8238 (3)	−0.2282 (9)	0.3586 (3)	0.0365 (8)	
C5	0.8196 (3)	−0.0934 (10)	0.2676 (3)	0.0381 (9)	
H5	0.871190	−0.135533	0.233150	0.046*	
C6	0.7376 (3)	0.1061 (9)	0.2273 (3)	0.0371 (9)	
H6	0.734210	0.204031	0.165071	0.045*	
C7	0.5754 (3)	0.3898 (9)	0.2214 (3)	0.0364 (9)	
C8	0.3804 (3)	0.9609 (11)	0.0786 (3)	0.0411 (10)	
H8	0.438654	0.903116	0.054610	0.049*	
C9	0.3047 (4)	1.1621 (10)	0.0206 (3)	0.0424 (10)	
H9	0.313962	1.243052	−0.040174	0.051*	
C10	0.2177 (3)	1.2428 (10)	0.0506 (3)	0.0389 (9)	
H10	0.165082	1.375120	0.010283	0.047*	
C11	0.2075 (3)	1.1243 (8)	0.1438 (3)	0.0341 (9)	
C12	0.1202 (3)	1.1733 (9)	0.1847 (3)	0.0371 (9)	
C13	0.1160 (3)	1.0509 (11)	0.2741 (3)	0.0402 (9)	
H13	0.057188	1.093775	0.299480	0.048*	
C14	0.1991 (4)	0.8605 (10)	0.3290 (3)	0.0435 (10)	
H14	0.196326	0.774460	0.391516	0.052*	
C15	0.2838 (3)	0.7987 (10)	0.2925 (3)	0.0411 (10)	
H15	0.339701	0.669393	0.329616	0.049*	
C16	0.2884 (3)	0.9272 (9)	0.1997 (3)	0.0343 (8)	
H1	0.463 (5)	0.632 (17)	0.231 (5)	0.09 (2)*	

### 2-Chloro-4-nitrobenzoic acid–5-nitroquinoline (I) Atomic displacement parameters (Å^2^)

**Table:**

	*U*^11^	*U*^22^	*U*^33^	*U*^12^	*U*^13^	*U*^23^
Cl1	0.0495 (5)	0.0641 (7)	0.0462 (5)	0.0116 (6)	0.0317 (4)	0.0062 (5)
O1	0.0504 (17)	0.0543 (19)	0.0481 (18)	0.0154 (16)	0.0296 (14)	0.0074 (15)
O2	0.0569 (18)	0.072 (3)	0.0509 (18)	0.0215 (19)	0.0349 (16)	0.0194 (17)
O3	0.0518 (17)	0.053 (2)	0.058 (2)	0.0071 (17)	0.0268 (15)	0.0135 (17)
O4	0.0453 (15)	0.051 (2)	0.0562 (18)	0.0121 (16)	0.0265 (14)	−0.0026 (15)
O5	0.0488 (16)	0.0386 (16)	0.0605 (18)	0.0045 (17)	0.0214 (14)	0.0071 (17)
O6	0.0395 (16)	0.075 (3)	0.059 (2)	0.0106 (18)	0.0262 (14)	0.0093 (19)
N1	0.0357 (15)	0.0351 (17)	0.0474 (18)	0.0017 (17)	0.0183 (13)	−0.0015 (17)
N2	0.0367 (16)	0.0413 (19)	0.0407 (18)	0.0012 (16)	0.0221 (14)	−0.0009 (15)
N3	0.0418 (18)	0.044 (2)	0.0441 (19)	0.0080 (17)	0.0230 (16)	−0.0009 (16)
C1	0.0354 (19)	0.0340 (19)	0.0375 (19)	−0.0036 (17)	0.0196 (16)	−0.0050 (16)
C2	0.0375 (17)	0.042 (2)	0.0383 (19)	−0.0025 (19)	0.0218 (15)	−0.0042 (18)
C3	0.040 (2)	0.040 (2)	0.039 (2)	0.0023 (19)	0.0194 (16)	0.0000 (18)
C4	0.0366 (19)	0.033 (2)	0.043 (2)	0.0024 (18)	0.0178 (16)	−0.0027 (17)
C5	0.0365 (19)	0.042 (2)	0.043 (2)	0.0039 (18)	0.0229 (17)	−0.0003 (18)
C6	0.0378 (19)	0.043 (2)	0.0368 (18)	−0.0002 (19)	0.0206 (16)	−0.0005 (18)
C7	0.0346 (18)	0.039 (2)	0.041 (2)	0.0002 (18)	0.0197 (16)	−0.0029 (17)
C8	0.0389 (19)	0.050 (2)	0.042 (2)	0.001 (2)	0.0235 (18)	−0.0018 (19)
C9	0.045 (2)	0.051 (3)	0.039 (2)	0.000 (2)	0.0238 (18)	0.0054 (18)
C10	0.041 (2)	0.042 (2)	0.037 (2)	−0.0007 (19)	0.0179 (16)	0.0021 (18)
C11	0.0347 (18)	0.036 (2)	0.0365 (18)	−0.0015 (17)	0.0181 (15)	−0.0025 (16)
C12	0.036 (2)	0.037 (2)	0.043 (2)	0.0049 (17)	0.0197 (17)	−0.0022 (17)
C13	0.0387 (18)	0.046 (2)	0.044 (2)	0.002 (2)	0.0254 (16)	−0.004 (2)
C14	0.045 (2)	0.053 (3)	0.041 (2)	0.003 (2)	0.0243 (17)	0.002 (2)
C15	0.041 (2)	0.047 (2)	0.042 (2)	0.009 (2)	0.0223 (17)	0.007 (2)
C16	0.0344 (17)	0.036 (2)	0.0374 (19)	0.0017 (17)	0.0187 (15)	0.0009 (16)

### 2-Chloro-4-nitrobenzoic acid–5-nitroquinoline (I) Geometric parameters (Å, º)

**Table:**

Cl1—C2	1.729 (4)	C5—C6	1.386 (6)
O1—C7	1.288 (5)	C5—H5	0.9500
O1—H1	1.02 (8)	C6—H6	0.9500
O2—C7	1.209 (5)	C8—C9	1.402 (7)
O3—N1	1.228 (5)	C8—H8	0.9500
O4—N1	1.218 (4)	C9—C10	1.365 (5)
O5—N3	1.228 (5)	C9—H9	0.9500
O6—N3	1.220 (4)	C10—C11	1.425 (5)
N1—C4	1.474 (5)	C10—H10	0.9500
N2—C8	1.320 (5)	C11—C16	1.415 (6)
N2—C16	1.370 (4)	C11—C12	1.425 (5)
N3—C12	1.474 (5)	C12—C13	1.359 (6)
C1—C2	1.390 (6)	C13—C14	1.406 (6)
C1—C6	1.405 (5)	C13—H13	0.9500
C1—C7	1.514 (6)	C14—C15	1.369 (5)
C2—C3	1.403 (6)	C14—H14	0.9500
C3—C4	1.380 (5)	C15—C16	1.413 (5)
C3—H3	0.9500	C15—H15	0.9500
C4—C5	1.372 (6)		
			
C7—O1—H1	109 (4)	O1—C7—C1	115.9 (4)
O4—N1—O3	123.9 (4)	N2—C8—C9	122.0 (4)
O4—N1—C4	117.9 (4)	N2—C8—H8	119.0
O3—N1—C4	118.2 (3)	C9—C8—H8	119.0
C8—N2—C16	119.5 (4)	C10—C9—C8	120.6 (4)
O6—N3—O5	124.0 (4)	C10—C9—H9	119.7
O6—N3—C12	117.1 (4)	C8—C9—H9	119.7
O5—N3—C12	118.9 (3)	C9—C10—C11	118.6 (4)
C2—C1—C6	118.2 (4)	C9—C10—H10	120.7
C2—C1—C7	126.9 (3)	C11—C10—H10	120.7
C6—C1—C7	114.8 (3)	C16—C11—C12	115.6 (3)
C1—C2—C3	120.7 (3)	C16—C11—C10	117.8 (3)
C1—C2—Cl1	124.3 (3)	C12—C11—C10	126.5 (4)
C3—C2—Cl1	115.0 (3)	C13—C12—C11	123.1 (4)
C4—C3—C2	118.4 (4)	C13—C12—N3	116.4 (3)
C4—C3—H3	120.8	C11—C12—N3	120.5 (4)
C2—C3—H3	120.8	C12—C13—C14	119.7 (3)
C5—C4—C3	122.9 (4)	C12—C13—H13	120.1
C5—C4—N1	118.9 (3)	C14—C13—H13	120.1
C3—C4—N1	118.2 (4)	C15—C14—C13	120.2 (4)
C4—C5—C6	117.9 (3)	C15—C14—H14	119.9
C4—C5—H5	121.0	C13—C14—H14	119.9
C6—C5—H5	121.0	C14—C15—C16	120.0 (4)
C5—C6—C1	121.8 (4)	C14—C15—H15	120.0
C5—C6—H6	119.1	C16—C15—H15	120.0
C1—C6—H6	119.1	N2—C16—C15	117.2 (4)
O2—C7—O1	124.2 (4)	N2—C16—C11	121.5 (4)
O2—C7—C1	119.9 (4)	C15—C16—C11	121.3 (3)
			
C6—C1—C2—C3	−0.4 (6)	C8—C9—C10—C11	1.8 (7)
C7—C1—C2—C3	−179.9 (4)	C9—C10—C11—C16	−0.4 (6)
C6—C1—C2—Cl1	−178.5 (3)	C9—C10—C11—C12	−177.1 (4)
C7—C1—C2—Cl1	2.1 (6)	C16—C11—C12—C13	2.9 (6)
C1—C2—C3—C4	0.5 (6)	C10—C11—C12—C13	179.7 (4)
Cl1—C2—C3—C4	178.7 (3)	C16—C11—C12—N3	−176.6 (4)
C2—C3—C4—C5	−1.0 (7)	C10—C11—C12—N3	0.2 (6)
C2—C3—C4—N1	−179.4 (4)	O6—N3—C12—C13	−34.6 (6)
O4—N1—C4—C5	3.3 (6)	O5—N3—C12—C13	144.6 (4)
O3—N1—C4—C5	−176.7 (4)	O6—N3—C12—C11	144.9 (4)
O4—N1—C4—C3	−178.1 (4)	O5—N3—C12—C11	−35.9 (6)
O3—N1—C4—C3	1.8 (6)	C11—C12—C13—C14	−1.7 (7)
C3—C4—C5—C6	1.3 (7)	N3—C12—C13—C14	177.8 (4)
N1—C4—C5—C6	179.7 (4)	C12—C13—C14—C15	0.0 (7)
C4—C5—C6—C1	−1.2 (6)	C13—C14—C15—C16	0.2 (7)
C2—C1—C6—C5	0.8 (6)	C8—N2—C16—C15	179.2 (4)
C7—C1—C6—C5	−179.7 (4)	C8—N2—C16—C11	0.2 (6)
C2—C1—C7—O2	−176.0 (4)	C14—C15—C16—N2	−177.9 (4)
C6—C1—C7—O2	4.5 (6)	C14—C15—C16—C11	1.1 (7)
C2—C1—C7—O1	4.2 (6)	C12—C11—C16—N2	176.4 (4)
C6—C1—C7—O1	−175.3 (4)	C10—C11—C16—N2	−0.7 (6)
C16—N2—C8—C9	1.3 (7)	C12—C11—C16—C15	−2.6 (6)
N2—C8—C9—C10	−2.4 (7)	C10—C11—C16—C15	−179.6 (4)

### 2-Chloro-4-nitrobenzoic acid–5-nitroquinoline (I) Hydrogen-bond geometry (Å, º)

*Cg*3 and *Cg*4 are the centroids of the C11–C16 ring and the N2/C8–C16 ring system, respectively.

**Table:**

*D*—H···*A*	*D*—H	H···*A*	*D*···*A*	*D*—H···*A*
O1—H1···N2	1.02 (8)	1.58 (7)	2.585 (5)	168 (7)
C8—H8···O2^i^	0.95	2.59	3.174 (6)	120
C9—H9···O2^i^	0.95	2.56	3.152 (6)	120
C13—H13···O4^ii^	0.95	2.52	3.289 (6)	138
N3—O5···*Cg*3^iii^	1.23 (1)	3.06 (1)	3.724 (4)	113 (1)
N3—O5···*Cg*4^iii^	1.23 (1)	3.25 (1)	4.118 (4)	128 (1)

Symmetry codes: (i) −*x*+1, *y*+1/2, −*z*; (ii) *x*−1, *y*+2, *z*; (iii) *x*, *y*+1, *z*.

### 5-Chloro-2-nitrobenzoic acid–5-nitroquinoline (1/1) (II) Crystal data

**Table:**

C_7_H_4_ClNO_4_·C_9_H_6_N_2_O_2_	*Z* = 2
*M**_r_* = 375.72	*F*(000) = 384.00
Triclinic, *P*1	*D*_x_ = 1.596 Mg m^−^^3^
*a* = 7.6682 (6) Å	Mo *K*α radiation, λ = 0.71075 Å
*b* = 8.6515 (8) Å	Cell parameters from 7062 reflections
*c* = 12.8609 (10) Å	θ = 3.1–30.1°
α = 79.170 (3)°	µ = 0.29 mm^−^^1^
β = 78.968 (2)°	*T* = 190 K
γ = 70.394 (3)°	Block, colorless
*V* = 781.80 (11) Å^3^	0.26 × 0.20 × 0.18 mm

### 5-Chloro-2-nitrobenzoic acid–5-nitroquinoline (1/1) (II) Data collection

**Table:**

Rigaku R-AXIS RAPID diffractometer	3075 reflections with *I* > 2σ(*I*)
Detector resolution: 10.000 pixels mm^-1^	*R*_int_ = 0.055
ω scans	θ_max_ = 30.0°, θ_min_ = 3.1°
Absorption correction: numerical (NUMABS; Higashi, 1999)	*h* = −10→10
*T*_min_ = 0.933, *T*_max_ = 0.950	*k* = −12→12
9772 measured reflections	*l* = −18→16
4502 independent reflections	

### 5-Chloro-2-nitrobenzoic acid–5-nitroquinoline (1/1) (II) Refinement

**Table:**

Refinement on *F*^2^	Primary atom site location: structure-invariant direct methods
Least-squares matrix: full	Secondary atom site location: difference Fourier map
*R*[*F*^2^ > 2σ(*F*^2^)] = 0.052	Hydrogen site location: mixed
*wR*(*F*^2^) = 0.148	H atoms treated by a mixture of independent and constrained refinement
*S* = 1.09	*w* = 1/[σ^2^(*F*_o_^2^) + (0.077*P*)^2^] where *P* = (*F*_o_^2^ + 2*F*_c_^2^)/3
4502 reflections	(Δ/σ)_max_ < 0.001
239 parameters	Δρ_max_ = 0.42 e Å^−^^3^
0 restraints	Δρ_min_ = −0.39 e Å^−^^3^

### 5-Chloro-2-nitrobenzoic acid–5-nitroquinoline (1/1) (II) Special details

**Table:**

Geometry. All esds (except the esd in the dihedral angle between two l.s. planes) are estimated using the full covariance matrix. The cell esds are taken into account individually in the estimation of esds in distances, angles and torsion angles; correlations between esds in cell parameters are only used when they are defined by crystal symmetry. An approximate (isotropic) treatment of cell esds is used for estimating esds involving l.s. planes.
Refinement. Reflections were merged by SHELXL according to the crystal class for the calculation of statistics and refinement. _reflns_Friedel_fraction is defined as the number of unique Friedel pairs measured divided by the number that would be possible theoretically, ignoring centric projections and systematic absences.

### 5-Chloro-2-nitrobenzoic acid–5-nitroquinoline (1/1) (II) Fractional atomic coordinates and isotropic or equivalent isotropic displacement parameters (Å^2^)

**Table:**

	*x*	*y*	*z*	*U*_iso_*/*U*_eq_	
Cl1	0.14420 (7)	0.28783 (7)	−0.10243 (4)	0.05199 (18)	
O1	0.27021 (17)	0.49296 (15)	0.23397 (9)	0.0398 (3)	
O2	0.49601 (18)	0.27221 (17)	0.29240 (9)	0.0464 (3)	
O3	0.7904 (2)	0.35306 (18)	0.13902 (11)	0.0517 (4)	
O4	0.91612 (19)	0.09724 (19)	0.11445 (12)	0.0560 (4)	
O5	−0.2211 (2)	0.9235 (2)	0.73000 (11)	0.0588 (4)	
O6	−0.3859 (2)	1.13276 (19)	0.63614 (12)	0.0585 (4)	
N1	0.78959 (19)	0.22747 (19)	0.10917 (11)	0.0375 (3)	
N2	0.18718 (18)	0.57290 (17)	0.42980 (10)	0.0304 (3)	
N3	−0.2703 (2)	0.99626 (19)	0.64474 (12)	0.0363 (3)	
C1	0.4473 (2)	0.31055 (18)	0.11065 (11)	0.0280 (3)	
C2	0.6260 (2)	0.2358 (2)	0.06195 (12)	0.0311 (3)	
C3	0.6607 (3)	0.1738 (2)	−0.03429 (13)	0.0394 (4)	
H3	0.785039	0.121041	−0.064631	0.047*	
C4	0.5112 (3)	0.1900 (2)	−0.08550 (13)	0.0405 (4)	
H4	0.531267	0.149424	−0.151912	0.049*	
C5	0.3327 (2)	0.2660 (2)	−0.03861 (12)	0.0347 (4)	
C6	0.2973 (2)	0.3264 (2)	0.05903 (12)	0.0322 (3)	
H6	0.172692	0.377559	0.089801	0.039*	
C7	0.4089 (2)	0.3576 (2)	0.22188 (12)	0.0289 (3)	
C8	0.2766 (2)	0.4942 (2)	0.51114 (13)	0.0338 (4)	
H8	0.379851	0.397342	0.501163	0.041*	
C9	0.2254 (2)	0.5479 (2)	0.61268 (13)	0.0360 (4)	
H9	0.295494	0.488936	0.669132	0.043*	
C10	0.0752 (2)	0.6842 (2)	0.62990 (12)	0.0329 (4)	
H10	0.039659	0.720138	0.698459	0.039*	
C11	−0.0277 (2)	0.77237 (19)	0.54485 (11)	0.0262 (3)	
C12	−0.1866 (2)	0.9174 (2)	0.54633 (12)	0.0287 (3)	
C13	−0.2716 (2)	0.9935 (2)	0.45823 (13)	0.0335 (3)	
H13	−0.376955	1.090140	0.462933	0.040*	
C14	−0.2037 (2)	0.9294 (2)	0.36000 (13)	0.0364 (4)	
H14	−0.264213	0.981948	0.298918	0.044*	
C15	−0.0520 (2)	0.7928 (2)	0.35311 (12)	0.0335 (4)	
H15	−0.005289	0.751029	0.286601	0.040*	
C16	0.0378 (2)	0.71153 (19)	0.44377 (11)	0.0269 (3)	
H1	0.249 (4)	0.510 (4)	0.310 (3)	0.097 (9)*	

### 5-Chloro-2-nitrobenzoic acid–5-nitroquinoline (1/1) (II) Atomic displacement parameters (Å^2^)

**Table:**

	*U*^11^	*U*^22^	*U*^33^	*U*^12^	*U*^13^	*U*^23^
Cl1	0.0591 (3)	0.0735 (4)	0.0357 (3)	−0.0300 (3)	−0.0154 (2)	−0.0106 (2)
O1	0.0383 (7)	0.0413 (7)	0.0295 (6)	0.0079 (5)	−0.0075 (5)	−0.0150 (5)
O2	0.0500 (8)	0.0468 (8)	0.0289 (6)	0.0086 (6)	−0.0146 (5)	−0.0075 (5)
O3	0.0544 (8)	0.0521 (9)	0.0544 (8)	−0.0177 (7)	−0.0210 (7)	−0.0056 (7)
O4	0.0337 (7)	0.0574 (10)	0.0559 (8)	0.0116 (6)	−0.0050 (6)	−0.0061 (7)
O5	0.0689 (10)	0.0644 (10)	0.0325 (7)	−0.0043 (8)	0.0003 (7)	−0.0192 (7)
O6	0.0579 (9)	0.0479 (9)	0.0567 (9)	0.0053 (7)	−0.0009 (7)	−0.0234 (7)
N1	0.0296 (7)	0.0450 (9)	0.0289 (7)	−0.0034 (6)	−0.0018 (5)	−0.0007 (6)
N2	0.0294 (6)	0.0313 (7)	0.0293 (6)	−0.0064 (5)	−0.0030 (5)	−0.0079 (5)
N3	0.0347 (8)	0.0400 (8)	0.0355 (7)	−0.0129 (6)	0.0037 (6)	−0.0147 (6)
C1	0.0310 (8)	0.0252 (7)	0.0227 (7)	−0.0023 (6)	−0.0029 (6)	−0.0038 (6)
C2	0.0302 (8)	0.0287 (8)	0.0288 (7)	−0.0030 (6)	−0.0037 (6)	−0.0022 (6)
C3	0.0409 (9)	0.0378 (9)	0.0310 (8)	−0.0029 (7)	0.0048 (7)	−0.0111 (7)
C4	0.0538 (11)	0.0396 (10)	0.0275 (8)	−0.0120 (8)	−0.0001 (7)	−0.0130 (7)
C5	0.0455 (10)	0.0364 (9)	0.0252 (7)	−0.0145 (7)	−0.0090 (7)	−0.0040 (7)
C6	0.0324 (8)	0.0343 (8)	0.0262 (7)	−0.0045 (7)	−0.0042 (6)	−0.0057 (6)
C7	0.0285 (7)	0.0313 (8)	0.0244 (7)	−0.0035 (6)	−0.0052 (6)	−0.0067 (6)
C8	0.0298 (8)	0.0339 (9)	0.0371 (8)	−0.0069 (7)	−0.0079 (6)	−0.0054 (7)
C9	0.0365 (9)	0.0405 (9)	0.0321 (8)	−0.0107 (7)	−0.0129 (7)	−0.0016 (7)
C10	0.0356 (8)	0.0410 (9)	0.0251 (7)	−0.0134 (7)	−0.0057 (6)	−0.0078 (7)
C11	0.0276 (7)	0.0294 (8)	0.0242 (7)	−0.0121 (6)	−0.0023 (5)	−0.0051 (6)
C12	0.0279 (7)	0.0305 (8)	0.0283 (7)	−0.0109 (6)	0.0021 (6)	−0.0080 (6)
C13	0.0286 (8)	0.0301 (8)	0.0387 (9)	−0.0072 (6)	−0.0023 (6)	−0.0033 (7)
C14	0.0355 (9)	0.0393 (9)	0.0305 (8)	−0.0070 (7)	−0.0089 (7)	0.0007 (7)
C15	0.0368 (9)	0.0382 (9)	0.0237 (7)	−0.0081 (7)	−0.0054 (6)	−0.0052 (6)
C16	0.0268 (7)	0.0298 (8)	0.0245 (7)	−0.0093 (6)	−0.0027 (6)	−0.0045 (6)

### 5-Chloro-2-nitrobenzoic acid–5-nitroquinoline (1/1) (II) Geometric parameters (Å, º)

**Table:**

Cl1—C5	1.7351 (17)	C4—H4	0.9500
O1—C7	1.3022 (18)	C5—C6	1.395 (2)
O1—H1	1.00 (3)	C6—H6	0.9500
O2—C7	1.2098 (19)	C8—C9	1.409 (2)
O3—N1	1.2207 (19)	C8—H8	0.9500
O4—N1	1.2158 (19)	C9—C10	1.362 (2)
O5—N3	1.217 (2)	C9—H9	0.9500
O6—N3	1.216 (2)	C10—C11	1.419 (2)
N1—C2	1.470 (2)	C10—H10	0.9500
N2—C8	1.312 (2)	C11—C12	1.426 (2)
N2—C16	1.365 (2)	C11—C16	1.431 (2)
N3—C12	1.4829 (19)	C12—C13	1.362 (2)
C1—C2	1.385 (2)	C13—C14	1.411 (2)
C1—C6	1.391 (2)	C13—H13	0.9500
C1—C7	1.5085 (19)	C14—C15	1.355 (2)
C2—C3	1.385 (2)	C14—H14	0.9500
C3—C4	1.384 (3)	C15—C16	1.415 (2)
C3—H3	0.9500	C15—H15	0.9500
C4—C5	1.378 (2)		
			
C7—O1—H1	107.9 (18)	O1—C7—C1	113.35 (13)
O4—N1—O3	124.32 (16)	N2—C8—C9	122.56 (15)
O4—N1—C2	118.09 (15)	N2—C8—H8	118.7
O3—N1—C2	117.58 (14)	C9—C8—H8	118.7
C8—N2—C16	119.04 (13)	C10—C9—C8	119.96 (15)
O6—N3—O5	122.72 (14)	C10—C9—H9	120.0
O6—N3—C12	117.87 (15)	C8—C9—H9	120.0
O5—N3—C12	119.41 (15)	C9—C10—C11	119.64 (14)
C2—C1—C6	118.14 (13)	C9—C10—H10	120.2
C2—C1—C7	122.53 (13)	C11—C10—H10	120.2
C6—C1—C7	118.97 (13)	C10—C11—C12	128.17 (13)
C1—C2—C3	122.79 (15)	C10—C11—C16	116.46 (14)
C1—C2—N1	120.19 (13)	C12—C11—C16	115.35 (14)
C3—C2—N1	116.94 (14)	C13—C12—C11	122.78 (13)
C4—C3—C2	118.91 (16)	C13—C12—N3	115.36 (14)
C4—C3—H3	120.5	C11—C12—N3	121.86 (14)
C2—C3—H3	120.5	C12—C13—C14	120.30 (15)
C5—C4—C3	118.91 (14)	C12—C13—H13	119.9
C5—C4—H4	120.5	C14—C13—H13	119.9
C3—C4—H4	120.5	C15—C14—C13	119.77 (15)
C4—C5—C6	122.30 (15)	C15—C14—H14	120.1
C4—C5—Cl1	119.31 (12)	C13—C14—H14	120.1
C6—C5—Cl1	118.40 (13)	C14—C15—C16	120.81 (14)
C1—C6—C5	118.93 (15)	C14—C15—H15	119.6
C1—C6—H6	120.5	C16—C15—H15	119.6
C5—C6—H6	120.5	N2—C16—C15	116.71 (13)
O2—C7—O1	124.62 (13)	N2—C16—C11	122.30 (14)
O2—C7—C1	121.98 (14)	C15—C16—C11	120.99 (14)
			
C6—C1—C2—C3	−1.3 (3)	C8—C9—C10—C11	−0.6 (2)
C7—C1—C2—C3	171.77 (16)	C9—C10—C11—C12	−179.44 (15)
C6—C1—C2—N1	175.37 (14)	C9—C10—C11—C16	−1.2 (2)
C7—C1—C2—N1	−11.5 (2)	C10—C11—C12—C13	178.45 (16)
O4—N1—C2—C1	135.40 (16)	C16—C11—C12—C13	0.2 (2)
O3—N1—C2—C1	−45.7 (2)	C10—C11—C12—N3	−1.3 (2)
O4—N1—C2—C3	−47.7 (2)	C16—C11—C12—N3	−179.55 (13)
O3—N1—C2—C3	131.17 (17)	O6—N3—C12—C13	−11.0 (2)
C1—C2—C3—C4	1.4 (3)	O5—N3—C12—C13	169.25 (16)
N1—C2—C3—C4	−175.35 (16)	O6—N3—C12—C11	168.68 (15)
C2—C3—C4—C5	−0.6 (3)	O5—N3—C12—C11	−11.0 (2)
C3—C4—C5—C6	−0.3 (3)	C11—C12—C13—C14	0.2 (2)
C3—C4—C5—Cl1	179.69 (14)	N3—C12—C13—C14	179.91 (14)
C2—C1—C6—C5	0.4 (2)	C12—C13—C14—C15	−0.8 (3)
C7—C1—C6—C5	−172.98 (15)	C13—C14—C15—C16	1.1 (3)
C4—C5—C6—C1	0.4 (3)	C8—N2—C16—C15	179.18 (15)
Cl1—C5—C6—C1	−179.59 (12)	C8—N2—C16—C11	−1.4 (2)
C2—C1—C7—O2	−37.4 (2)	C14—C15—C16—N2	178.69 (15)
C6—C1—C7—O2	135.67 (18)	C14—C15—C16—C11	−0.7 (2)
C2—C1—C7—O1	145.23 (15)	C10—C11—C16—N2	2.2 (2)
C6—C1—C7—O1	−41.7 (2)	C12—C11—C16—N2	−179.29 (13)
C16—N2—C8—C9	−0.5 (2)	C10—C11—C16—C15	−178.39 (14)
N2—C8—C9—C10	1.5 (3)	C12—C11—C16—C15	0.1 (2)

### 5-Chloro-2-nitrobenzoic acid–5-nitroquinoline (1/1) (II) Hydrogen-bond geometry (Å, º)

**Table:**

*D*—H···*A*	*D*—H	H···*A*	*D*···*A*	*D*—H···*A*
O1—H1···N2	0.99 (4)	1.66 (4)	2.6405 (17)	169 (3)
C3—H3···O4^i^	0.95	2.49	3.408 (3)	162
C10—H10···O3^ii^	0.95	2.54	3.254 (2)	132
C13—H13···O2^iii^	0.95	2.59	3.190 (2)	121
C14—H14···O2^iii^	0.95	2.56	3.173 (2)	122

Symmetry codes: (i) −*x*+2, −*y*, −*z*; (ii) −*x*+1, −*y*+1, −*z*+1; (iii) *x*−1, *y*+1, *z*.
